# Peri-Operative Fall in Serum Albumin Levels Correlate Well With Outcomes in Children Undergoing Emergency Abdominal Surgery: A Prospective Study From a Resource-Limited Setting

**DOI:** 10.7759/cureus.24960

**Published:** 2022-05-13

**Authors:** Rafey A Rahman, Muniba Alim, Sachit Anand

**Affiliations:** 1 Pediatric Surgery, Uttar Pradesh University of Medical Sciences, Etawah, IND; 2 Pediatrics, Uttar Pradesh University of Medical Sciences, Etawah, IND; 3 Pediatric Surgery, All India Institute of Medical Sciences, New Delhi, IND

**Keywords:** emergency, abdominal surgery, laparotomy, acute abdomen, perforation peritonitis, serum albumin, complications

## Abstract

Background

Albumin is a negative acute-phase protein as its levels fall after injury, sepsis, and surgical stress. A review of the literature suggests that serum albumin level drops rapidly after surgery in adults and correlates well with the outcomes. However, there is limited data on the use of peri-operative fall in serum albumin levels as an outcome predictor in children undergoing emergency abdominal surgeries. We aim to investigate the correlation between the degree of peri-operative fall in serum albumin levels and the outcomes in children undergoing emergent abdominal surgeries.

Materials and methods

This prospective study included all children aged 2-15 years undergoing emergent abdominal surgeries between January 2019 to June 2020 at our center. Preoperative serum albumin level (A1) was recorded for all children. Postoperative day 0 serum albumin level (A2) was sent 4-6 hours following the surgery. The degree of peri-operative fall in serum albumin level (∆A) was calculated by subtracting A2 from A1. Patients were then grouped on the basis of ∆ A, i.e. groups 1 and 2 with ∆ A < 0.5 and ≥ 0.5 gm/dl respectively. Additional data like diagnosis, surgical procedure, duration of surgery, complications, and length of hospital stay were also recorded. Recorded parameters in group 1 were then compared to group 2 statistically.

Results

Fifty-six children (male to female ratio {M:F} = 1.5:1), who met the inclusion criteria during the study period, were included in the study. Groups 1 and 2 comprised 38 and 18 children respectively. The postoperative serum albumin levels were significantly lower in group 2 (p = 0.0005). Duration of surgery was significantly higher in group 2 (p = 0.0474). Complications and length of hospital stay were significantly higher in group 2 (p = 0.0107 and p = 0.0375 respectively).

Conclusion

The present study evaluated the fall in peri-operative serum albumin level (∆A) in children undergoing emergent abdominal surgery as a marker of stress. Higher values of ∆A (≥ 0.5 gm/dl) depicted a significant correlation with complications requiring re-laparotomy and a longer length of hospital stay.

## Introduction

Serum albumin, the most abundant plasma protein, is considered to be the best parameter of nutritional assessment [1]. It represents 60% of the total plasma proteins and is responsible for maintaining plasma oncotic pressures. In addition, it also acts as a binding site for many molecules including fatty acids, bilirubin, metals, hormones, and certain drugs. It plays a key role in wound healing [1]. Albumin’s long biological half-life (18-20 days) along with its broad distribution prevents any nutritional changes to be rapidly reflected in its serum levels [1]. In addition to malnutrition, serum albumin levels are also influenced by other non-nutritional factors like infection, inflammation, liver diseases, and fluid retention [2]. Albumin is a negative acute-phase protein as its levels fall after injury, sepsis, and surgical stress [2]. Rhoads and Alexander, in 1955, identified hypoalbuminemia as a risk factor in surgical patients [3]. Since then, various studies have depicted that low serum albumin levels are associated with unfavorable outcomes, higher complication rates, longer hospital stay, and increased mortality among surgical patients [4,5].

A review of the literature suggests that serum albumin level drops rapidly after surgery in adults and correlates well with outcomes in esophageal [6], oral cancer [7], abdominal [8], and pancreatic [9] surgeries. During abdominal surgery, around 6-24% of the circulating plasma protein leaks into the peritoneal cavity [10]. A study by Gibbs et al., using a multiple regression model, demonstrated that postoperative day 1 serum albumin level was an independent predictor of postoperative complications [11]. Although few studies [1,12] have examined postoperative serum albumin levels in children after cardiac surgeries, there is a paucity of data on peri-operative serum albumin levels in children undergoing emergency abdominal surgeries. Therefore, in this study, we aim to investigate the correlation between the degree of peri-operative fall in serum albumin levels and the outcomes in children undergoing emergent abdominal surgeries. We hypothesize that a fall in the level of serum albumin following emergency abdominal surgery is a marker of surgical stress, and can replace costly biomarkers like C-reactive protein (CRP), especially in resource-challenged nations. In addition, the utilization of the fall in the level of serum albumin (and not merely the postoperative absolute value) makes this parameter more robust and resistant to fluctuations due to various perioperative factors.

## Materials and methods

Study setting and design

This prospective study was conducted at a tertiary care center located in the rural part of India. The study was conducted jointly by the Department of Pediatric Surgery and Pediatrics at the lead author’s institution after clearance from the Institutional Ethics Committee (IEC no:228/2020). Written informed consent for participation was taken from the parents at the time of recruitment. Children, aged 2 years to 15 years, who underwent emergent abdominal surgeries between January 2019 to June 2020 were included. Those who had surgeries for the abdomen along with other parts of the body in the same setting (polytrauma), malignancy, chronic liver diseases, and hematological diseases were excluded from the study.

All included children were admitted from the Emergency Room and stabilized. None of the children required albumin infusion during stabilization. All of them had a uniform fasting duration of 2-4 hours before surgery. During this period, essential hematological, biochemical, and radiological (X‑ray, ultrasound, and computed tomography as per the departmental protocol) investigations were performed. Albumin estimation was done by spectrophotometry. Preoperative serum albumin level (A1) was recorded for all children. As per the departmental protocol, we defined hypoalbuminemia as serum albumin < 3.4 g/dl and anemia as hemoglobin < 10 g/dl. All surgeries were performed by a single surgeon (RA) as per the departmental protocols. Postoperative day 0 serum albumin level (A2) was sent 4-6 hours following the surgery. The degree of peri-operative fall in the serum albumin level (∆A) was calculated by subtracting A2 from A1 (∆A = A1 − A2). Patients were then grouped into two based on the ∆A values. Groups 1 and 2 included children with ∆A < 0.5 and ≥ 0.5 g/dl, respectively. Additional data like diagnosis, surgical procedure, complications, and length of hospital stay were also recorded. The severity of the postoperative complications was categorized as per the Dindo-Clavien system [13]; grades I & II were considered minor complications and III & IV were considered major complications. All the recorded parameters were compared among the children of groups 1 and 2.

Statistical analysis

Data were entered in the Microsoft Excel sheet, and data consistency was checked by double entry. Data were analyzed using the SPSS software (Statistical Package for the Social Sciences, version 17.0, SPSS Inc., Chicago, USA). Qualitative data were represented in number and percentage, and quantitative data were represented in mean ± standard deviation (SD) (where t-test was applied) or median (maximum, minimum) (where the rank-sum test was applied). Continuous variables were compared between the groups by t-test/rank-sum test. Association between the categorical variables was tested using the chi-square test/Fisher’s exact test. A p-value < 0.05 was considered statistically significant.

## Results

A total of 56 children, who met the inclusion criteria during the study period, were included in the study. Out of these, 34 were males and 22 were females (M:F = 1.5:1). The mean age of the cohort was 8.57 years (Range: 2 years to 15 years). The mean preoperative hemoglobin level of the cohort was 9.42 g/dl (Range: 6.5 g/dl to 12.6 g/dl) with 62.5% (35/56) of the children having anemia at the time of admission. The mean preoperative serum albumin level (A1) of the cohort was 3.3 g/dl (range: 2.8 g/dl to 4.1 g/dl) with 57.14% (32/56) of the children (57.14%) having hypoalbuminemia on admission. The mean postoperative serum albumin level (A2) of the cohort was 3 g/dl (range: 2.5 g/dl to 3.7 g/dl). The mean fall in postoperative serum albumin level (∆A) of the cohort was 0.34 g/dl (range: 0 to 0.7 g/dl). Fifteen out of 56 children (26.78%) in the cohort developed postoperative complications. Surgical site infection, seen in 8 out of 15 children (53.33 %), was the most common complication. The mean length of hospital stay of the cohort was 6.67 days (range: 3 days to 12 days).

Group 1 (∆ A < 0.5 g/dl) comprised 38 children (M:F = 1.4:1). The mean age in this group was 8.13 years (range: 2 years to 14 years) (Table 1). The indications of surgery (Table 2) were intestinal obstruction (44.73; 17/38), acute abdomen (34.21; 13/38), and miscellaneous (21.06; 8/38). The mean preoperative hemoglobin level in this group was 9.49 g/dl (range: 7.1 g/dl to 11.2 g/dl) with 63.15% (24/38) of the children having anemia at the time of admission. The mean preoperative serum albumin level (A1) in this group was 3.28 g/dl (range: 2.8 g/dl to 3.9 g/dl) with 57.89% (22/38) of the children having hypoalbuminemia on admission. The mean postoperative serum albumin level (A2) in this group was 3.03 g/dl (range: 2.6 g/dl to 3.7 g/dl). The mean perioperative fall in serum albumin level (∆A ) was 0.25 g/dl (range: 0 to 0.4 g/dl). The average duration of surgery in this group was 90.26 min (range: 45 min to 130 min). Six out of 38 children (15.78 %) developed postoperative complications. As per the Dindo-Clavien severity system [13], there were 3 grade II complications and 3 grade III complications in this group (Table 3). The mean length of hospital stay in this group of children was 6.63 days (range: 3 days to 12 days).

**Table 1:** Comparison of various quantitative variables in group 1 (∆ A < 0.5 gm/dl) and group 2 (∆ A ≥ 0.5 gm/dl) *p-value calculated, p-value <0.05 is significant, A1: Pre-operative Serum Albumin levels, A2: Post-operative serum albumin levels, ∆ A: Fall in serum albumin levels post-operatively (A1 – A2)

Variables	Groups	N	Mean ± SD	Median (min, max)	p-value
Age (years)	Group 1	38	8.13 ± 3.61*	7 (2,14)	0.088
	Group 2	18	9.5 ± 3.11*	9.5 (3,15)	
Haemoglobin at admission (g/dl)	Group 1	38	9.49 ± 1.06*	9.5 (2,14)	0.496
	Group 2	18	9.27 ± 1.37*	9.25 (6.5,12.6)	
A1 (g/dl)	Group 1	38	3.28 ± 0.28*	3.2 (2.8,3.9)	0.355
	Group 2	18	3.25 ± 0.29*	3.2 (2.8,3.8)	
A2 (g/dl)	Group 1	38	3.03 ± 0.3*	2.95 (2.6,3.7)	0.0005
	Group 2	18	2.72 ± 0.3*	2.7 (2.3,3.3)	
Duration of Surgery (min)	Group 1	38	90.26 ± 25.26*	100 (45,130)	0.047
	Group 2	18	102.5 ± 23.46*	110 (45,150)	
Length of Hospital stay (Days)	Group 1	38	6.63 ± 2.21*	6 (3,12)	0.037
	Group 2	18	7.83 ± 2.38*	8 (3,12)	

**Table 2:** Diagnosis and indications for surgery in group 1 and group 2

Diagnosis	Group 1 (N)	Group 2 (N)
Acute Abdomen		
Perforation Peritonitis	7	6
Appendicular Pathology	3	2
Perforated Meckel’s Diverticulum	3	0
Taruma	0	3
Total (A)	13	11
Intestinal Obstruction		
Adhesive Obstruction	6	2
Omphalomesentric duct remnant	3	1
Intussusception	4	1
Worm obstruction	2	0
Obstructed Umbilical Hernia	1	0
Obstructed Inguinal Hernia	1	1
Total (B)	17	5
Miscellaneous		
Primary peritonitis	2	0
Psoas abscess	2	1
Liver abscess	3	0
Splenic abscess	1	1
Total (C)	8	2
Grand Total (A + B + C)	38	18

**Table 3:** Complications in group 1 and group 2

Complications (Dindo-Clavien severity grading)	Group 1 (N)	Group 2 (N)	Total
Surgical site infection (II)	3	5	8
Anastomotic leak (III)	2	2	4
Wound dehiscence (III)	1	2	3
Total	6	9	15

Group 2 (∆ A ≥ 0.5 g/dl) comprised 18 children (M:F = 2:1). The mean age in this group was 9.5 years (range: 3 years to 15 years). The indications of surgery (shown in Table 2) were intestinal obstruction (27.77; 5/18), acute abdomen (61.11; 11/18), and miscellaneous (11.12; 2/18). The mean preoperative hemoglobin level in this group was 9.27 g/dl (range: 6.5 g/dl to 12.6 g/dl) with 38.88% (7/18) of the children having anemia at the time of admission. The mean preoperative serum albumin level (A1) in this group was 3.25 gm/dl (range: 2.8 g/dl to 3.8 g/dl) with 55.55% (10/18) of the children (55.55%) having hypoalbuminemia on admission. The mean postoperative serum albumin level (A2) in this group was 2.72 g/dl (range: 2.3 g/dl to 3.3 g/dl). The mean perioperative fall in serum albumin level (∆A) was 0.53 g/dl (range: 0.5 g/dl to 0.7 g/dl). The average duration of surgery in this group was 102.5 min (range: 45 min to 150 min). Nine out of 18 children (50%) in this group developed postoperative complications. As per the Dindo-Clavien severity system [13], there were 5 grade II complications and 4 grade III complications in this group (as shown in Table 3). The mean length of hospital stay in this group of children was 7.83 days (Range: 3 days to 12 days).

On comparing the A1 serum albumin levels among the two groups (shown in Table 1), no significant difference was observed. However, the A2 serum albumin levels were significantly lower in group 2 (p = 0.0005). Also, a significantly prolonged duration of surgery (p = 0.0474) and length of hospital stay (p = 0.0375) were seen in group 2 as compared to group 1. Similarly, the proportion of children developing complications (Table 4) was significantly higher (p = 0.0107) in group 2. There were no mortalities in either group.

**Table 4:** Comparison of complications between group 1 and group 2 *Fisher Exact Test, Significant p-value < 0.05

Variables	Group 1	Group 2	Total	*p-value
Complications	6 (10.18) [1.72]	9 (4.82) [3.62]	15	0.0107
No Complication	32 (27.82) [0.63]	9 (13.18) [1.32]	41	
Total	38	18	56	

## Discussion

The ideal marker for measuring the stress and predicting the outcomes following surgery must be available early, easy to measure, and inexpensive. The present study evaluated the fall in peri-operative serum albumin levels (∆A) in children undergoing emergent abdominal surgery as a marker of stress. The findings of this study suggest that ∆A in such scenarios accurately quantifies the magnitude of surgical stress and can be reliably used as a predictor for the outcomes. This marker can be used as early as within 4-6 hours following surgery to predict the outcomes and can help in initiating timely intervention to decrease morbidity.

Various mechanisms by which there occurs a fall in peri-operative serum albumin levels include hemodilution, altered metabolism, and redistribution into the third space due to leakage from the capillaries. This redistribution is responsible for a 75% fall in the peri-operative serum albumin and relates to the magnitude of the systemic inflammatory response [14]. Emergent abdominal surgeries induce an important metabolic stress response that results in leakage of circulating plasma proteins into the peritoneal cavity [11]. This stress response can be studied by measuring various pro-inflammatory markers like interleukin (IL)-6 within hours following surgery [15-18]. However, its cost and sophistication make its routine use impractical at a center located in resource-limited settings. CRP, another acute phase reactant, correlates well with the magnitude of surgery and is being used to measure postoperative inflammatory response [19-21]. However, its kinetics are slow and peaks are reached on a postoperative day three or four, thus limiting its use as a marker for predicting the outcome on the day of surgery. CRP is produced in the liver and its levels are markedly reduced in liver diseases and following liver surgeries; thus, further limiting its use as an early marker for predicting the outcomes following liver surgeries [21]. Therefore, serum albumin outscores these markers in resource-limited settings as it is easily available, incurs a low cost, and has early reporting.

In the present study majority of the children had anemia and hypoalbuminemia on admission. This can be attributed to the low socioeconomic status of their families resulting in malnutrition. Hypoalbuminemia on admission can also be due to the underlying disease (acute abdomen, intestinal obstruction) as it may have triggered a systemic inflammatory response. Ryan et al. have studied the changes in serum albumin levels on a postoperative day one following esophagectomy and found that low serum albumin was associated with increased morbidity, higher reoperation rates, and longer ICU stays [6]. Similarly, in the present study, the postoperative serum albumin level was significantly lower in group 2 (∆A ≥ 0.5 g/dl) and the children in this group had higher complications and longer duration of hospital stay. This can be explained by the fact that these children were already under stress because of their disease at the time of admission and underwent another surgical stress for the treatment of the disease. It was also observed that ∆A significantly correlated to the duration of surgery. The children in group 2 had a longer duration of surgeries; thus, indicating that fall in postoperative serum albumin levels also depends on the duration of surgical stress. Longer duration of surgery results in increased redistribution of plasma proteins and a higher amount of third space loss [10].

Another noteworthy finding in our study was that preoperative serum albumin levels showed no significant association with complications and length of hospital stay unlike other studies [4,5], but ∆A correlated well with them. This observation is similar to that depicted in various other studies [11,22]. Hence, the degree of fall in serum albumin level appears to be a better marker than baseline/preoperative serum albumin for predicting the postoperative outcomes in malnourished children, as the latter can be influenced by various factors, e.g. preoperative nutritional status. Furthermore, ∆A can be measured within hours following surgery [6,23]. This helps in the early identification of patients at risk of developing complications and adequate nutritional support can be instituted early in these children. Finally, the inexpensive serum albumin levels are routinely measured for all patients undergoing major surgeries. It can accurately detect the magnitude of stress following surgery and thus can serve as an appropriate marker to predict the outcomes in resource-limited settings.

The results of the present study should be interpreted within the context of a few limitations. First, the study had a small sample size and a short recruitment time. Second, the heterogeneous cohort also did not allow us to identify the standard cut-off value for the decline in albumin. Third, we have used one-time preoperative and postoperative serum albumin values. There is a definite chance of laboratory error during single-point estimations. Therefore, future studies need to focus on multiple estimations to rule out laboratory error(s). Nevertheless, in a limited cohort of patients undergoing emergent abdominal surgery, ∆A significantly correlates with the postoperative outcomes. However, our findings need to be tested in a larger cohort of patients, and the efficacy of ∆A needs to be compared with established markers like CRP before any definite conclusions are drawn.

## Conclusions

The present study evaluated the decline in peri-operative serum albumin level (∆A) in children undergoing emergent abdominal surgery as a marker of stress. ∆A correlated well with complications and length of hospital stay in these children. ∆A ≥ 0.5 g/dl was associated with a higher complication rate and a longer duration of hospital stay. Prolonged duration of surgery was also associated with a greater decline in peri-operative serum albumin levels in these children.
